# Eruptive Disseminated Spitz Nevi: A Case Report and Review of the Literature

**DOI:** 10.7759/cureus.47097

**Published:** 2023-10-16

**Authors:** Heba Y Alojail

**Affiliations:** 1 Dermatology, College of Medicine, King Faisal University, Al-Ahsa, SAU

**Keywords:** benign nevi, benign juvenile melanoma, spitz juvenile melanoma, spitz naevus, eruptive disseminated spitz nevi

## Abstract

Spitz naevus (SN) are benign melanocytic lesions, which are classified into solitary, agminated, or disseminated forms. The most common form is solitary SN, typically occurring on the face and extremities of children. However, the disseminated SN is a rare presentation that can be either rapid eruptive or non-eruptive. The eruptive disseminated Spitz naevus (EDSN) often develops between the ages of 10 and 20 and affects nearly the whole-body surface, beginning in the trunk and/or extremities. Without prior reports from the population of Saudi Arabia, this eruptive SN has reportedly impacted native Alaskans (Americans), Koreans, Pakistanis, African Americans, and Hispanics. Eruptive disseminated SNs are rare, with only 27 cases having been reported in the literature. This article presents another case of EDSN.

## Introduction

Spitz nevus (SN) is a benign melanocytic lesion classified into solitary, agminated, or disseminated forms. The most common form is solitary SN, typically occurring on the face and extremities of children. However, the disseminated SN is a rare presentation that can be either rapid eruptive or non-eruptive. The eruptive disseminated Spitz nevus (EDSN) often develops between the ages of 10 and 20 and affects nearly the whole-body surface, beginning in the trunk and/or extremities [1]. Without prior reports from the population of Saudi Arabia, EDSN has reportedly impacted native Alaskans (Americans) [2], Koreans [3], Pakistanis [4], African Americans [5], and Hispanics [6]. EDSN is rare, with only 27 cases having been reported in the literature [1]. This report presents another case of EDSN.

## Case presentation

A 14-year-old Saudi female, born through a cesarean section after a full-term pregnancy, without any medical history, developmentally appropriate for her age, and performing well in school presented with several painless, pigmented skin lesions. The first skin lesions appeared on her right thigh when she was seven years old, but over the past two years, they had been spreading quickly throughout her body, sparing only her palms and soles. They had a rapid progressive evolution in size and number. No history of convulsions, as well as no family history of similar lesions, was found. Otherwise, the patient was in good health.

**Figure 1:**
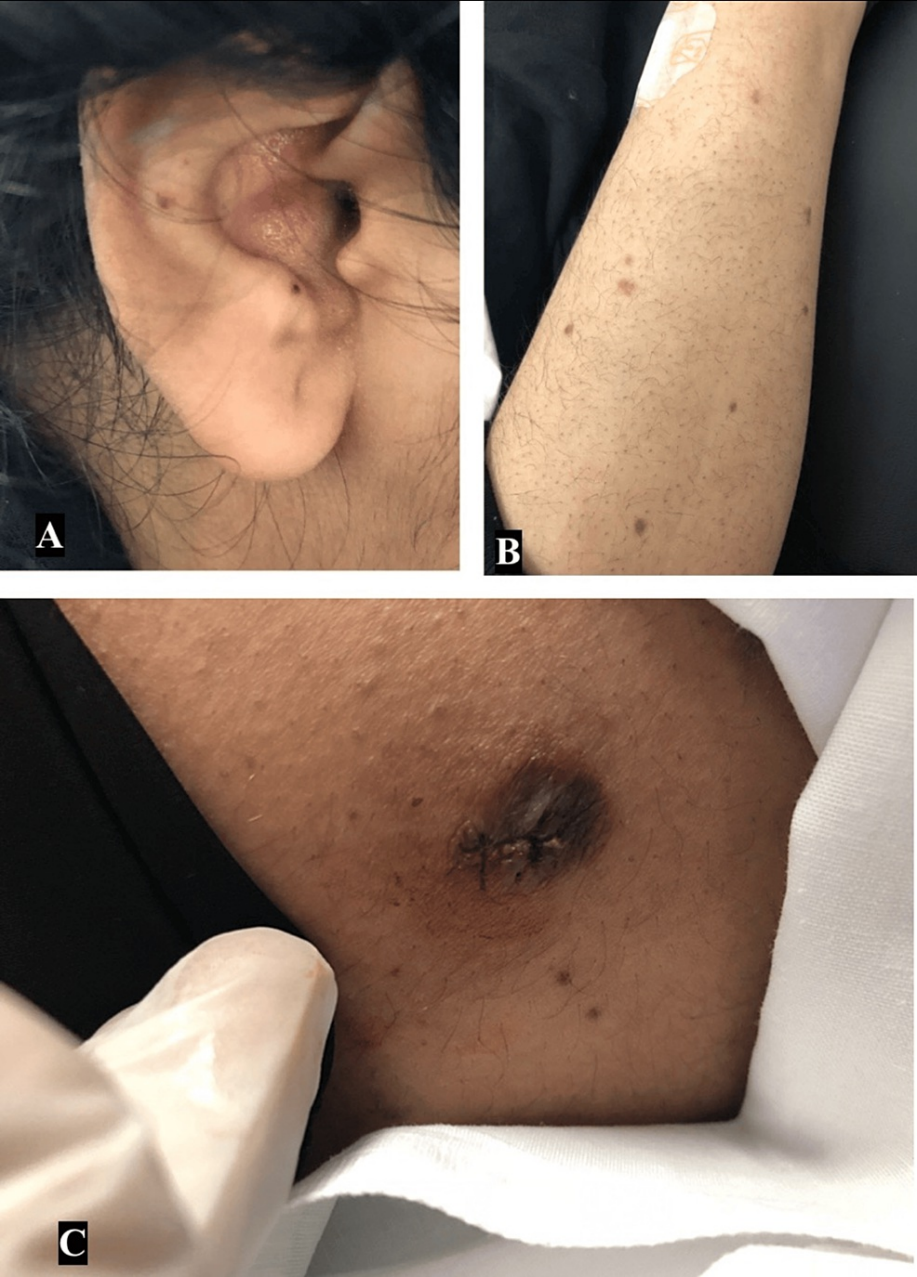
Multiple pink to brown papules on different parts of the body (A) Ear, (B) Arm, (C) Thighs

On physical examination, hundreds of well-defined dark-to-brown papules in different sizes were observed all over the ear, trunk, upper and lower limbs (Figures 1A-1B), and one single lesion over the right thigh (lateral aspect) (Figure 1C) in form of a well-defined brownish plaque. Palms and soles were spared.

No lymphadenopathy or hepatosplenomegaly was present. Incisional biopsies were performed for two pigmented lesions in order to histologically identify these lesions. The first biopsy was taken from the lateral aspect of the right thigh and the second biopsy from the anterior aspect of the right leg.

Histopathological examination of the biopsy of both lesions revealed a symmetric well-circumscribed proliferation of spindle and epithelioid melanocyte nests. Vertically oriented spindled melanocytes along rete (“bananas on the tree”) with no atypia and epidermal involvement were seen. Hyperplastic epidermis with high power (100 X) showed nests of spindled and epithelioid melanocytes with clefts between the nests. The epidermis's basement membrane was a dull pink, amphophilic substance that defines the Kamino body. Given these findings, a diagnosis of eruptive disseminated Spitz nevi was made. The patient did not have any treatment and she is periodically observed every six months.

## Discussion

EDSN was described in 1974 as juvenile eruptive melanoma by Wallace et al. [2]. Three clinical variants of SN have been described, solitary, agminated, and disseminated. The most common form is solitary SN, typically occurring on the face and extremities of children.

With over 40 cases recorded, the agminated or clustered variant, which occurs in localized clusters or a segmental distribution, is far less prevalent [7]. EDSN is rare, with only 27 cases having been reported in the literature [1]. The average age at which clinical symptoms appear is 21 years [8], however, the youngest patient in our report was 14 years old. Typically, the trunk and proximal limbs are where the lesions are found. Normally spared areas are the scalp, mucous membranes, palms, and soles; however, in one case, the palms and soles were also affected [9]. There have been four cases of spontaneous partial resolution [8].

The cause of EDSN is unknown, although several potential triggering conditions have been proposed, including the following: ultraviolet light exposure [10], pregnancy [11], chemotherapy [12], fever after tonsillectomy [13], Addison's disease [14] and perioperative stress [15,16]. No prior reports of EDSN in Saudi Arabia have ever been found in the literature.

The most important differential diagnosis is to distinguish between EDSN and metastatic malignant melanoma. Taking into account the age of the patient’s eruptive melanocytic nevi, urticaria pigmentosa, dysplastic nevi syndrome, and multiple juvenile xanthogranuloma need to be considered too. 

Simple complete surgical excision is the optimal treatment for a single Spitz nevi, but complete excision is not an option for EDSN due to the large number of lesions and the potential for significant complications. Of the reported cases, numerous remedy alternatives were tried, including electrocoagulation [5] liquid nitrogen [17], imiquimod, and laser ablation [18]. However, these options have not been successful. They have been used for aesthetic reasons, but in our opinion, non-surgical treatments are not recommended, as they might lead to cosmetically unacceptable results such as scarring post-inflammatory hypo/hyperpigmentation. Also, they do not allow accurate histological examination in the future because they are often followed by recurrence [2]. We recommend regular self-examination, full-body photography, dermoscopic follow-up for three to six months during the eruption phase, and immediate surgical excision of lesions suspected to be malignant. However, no malignant transformation of EDSN has been reported to date.

The longest follow-up for EDSN was 26 years with no malignant transformation in either the disseminated or the agminated forms of SN [13], so observation is an acceptable treatment option. However, it is important to recognize EDSN as a clinical entity to avoid misdiagnosis and potentially harmful or unnecessary therapy.

## Conclusions

Simple complete surgical excision is the best course of treatment for a single SN, but complete excision is not an option for EDSN due to the large number of lesions and the potential for significant complications. EDSN is uncommon, and the cause is unknown, but there are several potential triggering conditions. We advise regular self-examination, whole-body photography, dermoscopic follow-up for three to six months throughout the eruption period, and prompt surgical excision of lesions suspected of being malignant. To date, EDSN has not been observed to become malignant.
